# CRISPR-based resistance to grapevine virus A

**DOI:** 10.3389/fpls.2023.1296251

**Published:** 2023-12-04

**Authors:** Katarina P. Spencer, Johan T. Burger, Manuela Campa

**Affiliations:** Department of Genetics, Stellenbosch University, Stellenbosch, South Africa

**Keywords:** CRISPR/Cas, Cas13, CasRx, GIGS, virus interference, virus resistance, GVA interference

## Abstract

**Introduction:**

Grapevine (*Vitis vinifera*) is an important fruit crop which contributes significantly to the agricultural sector worldwide. Grapevine viruses are widespread and cause serious diseases which impact the quality and quantity of crop yields. More than 80 viruses plague grapevine, with RNA viruses constituting the largest of these. A recent extension to the clustered regularly interspaced, short palindromic repeat (CRISPR) armory is the Cas13 effector, which exclusively targets single-strand RNA. CRISPR/Cas has been implemented as a defense mechanism in plants, against both DNA and RNA viruses, by being programmed to directly target and cleave the viral genomes. The efficacy of the CRISPR/Cas tool in plants is dependent on efficient delivery of its components into plant cells.

**Methods:**

To this end, the aim of this study was to use the recent Cas13d variant from *Ruminococcus flavefaciens* (CasRx) to target the RNA virus, grapevine virus A (GVA). GVA naturally infects grapevine, but can infect the model plant *Nicotiana benthamiana*, making it a helpful model to study virus infection in grapevine. gRNAs were designed against the coat protein (*CP*) gene of GVA. *N. benthamiana* plants expressing CasRx were co-infiltrated with GVA, and with a tobacco rattle virus (TRV)-gRNA expression vector, harbouring a CP gRNA.

**Results and discussion:**

Results indicated more consistent GVA reductions, specifically gRNA CP-T2, which demonstrated a significant negative correlation with GVA accumulation, as well as multiple gRNA co-infiltrations which similarly showed reduced GVA titre. By establishing a virus-targeting defense system in plants, efficient virus interference mechanisms can be established and applied to major crops, such as grapevine.

## 1 Introduction

Global agriculture is under constant threat by plant diseases, which result in decreased yields and reduced quality. Plant viruses are responsible for numerous plant diseases and cause around 50% of the observed yield losses (Anderson et al., 2004; Boualem et al., 2016). The development and implementation of virus-resistant plant varieties would be a robust and sustainable solution to control plant virus diseases and viral infections (Varanda et al., 2021). Grapevine is a major fruit crop, which is cultivated globally and contributes to economic sectors worldwide, but is plagued by over 80 viruses (Fuchs, 2020). Grapevine virus A (GVA) is an RNA virus that replicates in the cytoplasm of the plant cell and is a member of the genus *Vitivirus* (Minafra et al., 1997; Martelli et al., 2007; Rampersad and Tennant, 2018). GVA naturally infects grapevine, but is also able to infect the model plant *Nicotiana benthamiana*, making it a helpful model for the study of viruses in herbaceous plants (Goszczynski and Jooste, 2003; Goodin et al., 2008; Ludman and Fátyol, 2019) By utilising an infectious clone of GVA, which has been modified to contain a region of the endogenous *N. benthamiana* phytoene desaturase (*NbPDS*) gene (pBINSN_GVA118_NbPDS) (Muruganantham et al., 2009), the establishment of an RNA virus-targeting system in *N. benthamiana* can be investigated. Adoption of *N. benthamiana* as a model plant is due to the time-consuming nature and low efficiency of both stable and transient transformation of grapevine. Indeed, generating a stably transformed grapevine plant expressing Cas13 that can be used for this type of investigation would take over a year (Dalla Costa et al., 2019; Campos et al., 2021). However, as the GVA infectious clone can infect *N. benthamiana*, the present study offers a proof of concept for the functionality of GVA-interference using Cas13.

The natural phenomenon of RNA silencing in plants was first discovered in 1990 and has since been characterized in several other eukaryotic organisms (Napoli et al., 1990; Romano and Macino, 1992; Gaudet et al., 1996). The role in antiviral immunity of RNA silencing and in particular of the sequence-specific RNA degradation process (post-transcriptional gene silencing [PTGS]), otherwise known as RNA interference (RNAi) (Petrov et al., 2019) was discovered, and has been successfully applied to target several plant virus species such as maize streak virus (MSV) (Shepherd et al., 2007), papaya ringspot virus (PRSV) (Fitch et al., 1992; Bau et al., 2003), potato virus Y (PVY) (Missiou et al., 2004; Tabassum et al., 2016) and tomato yellow leaf curl virus (TYLCV) (Fuentes et al., 2006), to name a few.

However, CRISPR/Cas has revolutionized antiviral strategies in plants by overcoming the shortfalls of methods such as RNAi (Echeverri et al., 2006; Cox et al., 2017; Wang et al., 2019), by offering precise and targeted genome editing (Wada et al., 2020; Zhu et al., 2020; Hryhorowicz et al., 2023). CRISPR/Cas technology has become popular within the scientific community due to its easy design, efficiency and flexibility, and has been harnessed to target both DNA and RNA viruses (Zaidi et al., 2016; Freije et al., 2019; Sandhya et al., 2020). The Cas13d subtype is smaller (less than 1000 aa, ~3 kb) than previous Cas13 effectors, while possessing efficient RNA interference abilities (Konermann et al., 2018; Mahas et al., 2019). The Cas13d variant from *Ruminococcus flavefaciens* (CasRx) has been harnessed for single-strand RNA-targeting and is an effective approach for RNA virus interference *in planta* (Mahas et al., 2019; Cao et al., 2021; Zhan et al., 2023).

Effective delivery and expression of CRISPR components in plants is necessary to achieve efficient and robust genome editing (Kuluev et al., 2019). Recently, the exploitation of plant viruses for the delivery of genome editing cassettes into plant cells has become popular, due to the easy manipulation of viral genomes and the ability of the virus to infect numerous plant species (Kalinina et al., 2020). Specifically, the RNA virus tobacco rattle virus (TRV) has been modified for transient and systemic expression of gRNAs in plants, and is an efficient system as it allows for the empirical testing and validation of multiple gRNAs simultaneously (Ali et al., 2015; Aman et al., 2018a; Kuluev et al., 2019; Aragonés et al., 2022; Son and Park, 2022; Uranga et al., 2023).

A recent, novel discovery within the CRISPR/Cas13 system is that in the absence of Cas13, a gRNA alone can elicit reductions in viral and endogenous plant RNA (Sharma et al., 2022). This mechanism, called guide-induced gene silencing (GIGS), was found to be dependent on sequence similarity of the gRNA and target RNA, and functions in a manner analogous to the endogenous RNA silencing or interference (RNAi) pathway. The discovery of GIGS provides a new avenue for targeted RNA reduction and virus interference methods in plants (Sharma et al., 2022).

The aim of this research was to induce virus interference by targeting the coat protein (CP) of GVA; by employing CRISPR/CasRx in *N. benthamiana*. Relative quantification of GVA transcripts were assessed to establish virus interference. The TRV-gRNA delivery system was used to deliver the CP-targeting gRNAs into transgenic *N. benthamiana* expressing the *CasRx* gene. Virus interference by GIGS was also assayed and compared to CRISPR/CasRx interference. Additionally, gRNA expression was quantified to compare expression levels from binary pCasRx : CP vectors and the TRV-gRNA delivery vectors.

## 2 Methods

### 2.1 Design of gRNAs

Three gRNAs were designed against the *CP* gene of GVA, using the software cas13design (https://cas13design.nygenome.org/, accessed on 15/01/2020) (Wessels et al., 2020; Guo et al., 2021). gRNAs were selected based on their rank, guide score, quartile, and off-target hits. *N. benthamiana* off-targets were assessed against the *N. benthamiana* transcriptome (https://sefapps02.qut.edu.au/blast/blast_link2.cgi, accessed on 15/01/2020). The secondary structure of the CasRx scaffold sequence and gRNA target [gaacccctaccaactggtcggggtttgaaacG (22-28nt target)] were assessed using the programs mFold (http://www.unafold.org/mfold/applications/rna-folding-form.php, accessed on 20/01/2020) and RNAfold (http://rna.tbi.univie.ac.at/cgi-bin/RNAWebSuite/RNAfold.cgi, accessed on 20/01/2020). The secondary structure of the target RNA was assessed with the same software, to ensure that the gRNA, specifically the seed region, was designed to target-accessible (single-strand or looped) regions (Bandaru et al., 2020). The gRNAs were designed to contain BbsI overhangs on their 5’-ends, for subsequent cloning with Golden Gate assembly. For cloning into the TRV2 vector, the gRNAs were designed to contain an XbaI overhang and CasRx scaffold sequence on their 5’-end and an XhoI overhang on their 3’-end (**Supplementary Table 1**).

### 2.2 Design and construction of CRISPR/CasRx constructs

Gibson Assembly (NEBuilder HiFi DNA Assembly Cloning Kit; NEB, USA) was used to modify two intermediate vectors: pJJB308, (Addgene; plasmid #107699) and pJJB296, (Addgene; plasmid #107691) to contain the inserts CasRx-gRNA promoter with scaffold sequence (~300bp), and the *CasRx* gene (~3kb), respectively, following the manufacturer’s protocol. Vector pJJB308 was digested with AarI, and the 2017bp vector fragment was recovered. Vector pJJB296 was digested with BamHI and HindIII, and the ~3.1kb vector fragment was recovered. PCR with a high-fidelity DNA polymerase (Phusion^®^ High-Fidelity DNA Polymerase; NEB, USA) was performed to amplify the desired insert fragments from pXR003:CasRx-gRNA-cloning-backbone, (Addgene plasmid #109053) and pXR001:EF1a-CasRx-2A-EGFP, (Addgene plasmid #109049) using specific primers (**Supplementary Table 2**). The purified insert and vector fragments were assembled according to the manufacturer’s instructions. The targets were ordered from IDT (Integrated DNA Technologies, USA) and synthesized as single strand DNA (ssDNA) oligonucleotides containing a BbsI (NEB, USA) overhang on the 5’-end (**Supplementary Table 1**). Adapter preparation consisted of annealing the ssDNA oligos in a reaction containing 1.5µL forward oligo (100µM), 1.5µL reverse oligo (100µM), 5µL 10X NEBuffer 2.1, and dH2O to a final volume of 50µL. The reactions were incubated in a thermocycler at 95°C for 4 minutes, followed by 70°C for 10 minutes. Reactions were transferred to a beaker containing 1L of H_2_O at 70°C and left to cool to room temperature. The pJJB308-CasRx-backbone was linearized with restriction enzyme BbsI (NEB, USA) and the ~2.3kb fragment was recovered. A ligation reaction containing the annealed oligos and linearized vector was set up using 1µL annealed oligos and 20ng linearized vector. The annealed gRNAs were cloned under the hU6 promoter, and upstream of the CasRx-gRNA scaffold. The vector maps of the Gibson-assembled intermediate vectors are shown in **Supplementary Figure 1**.

The final binary vector pCasRx : CP was constructed using a one-step Golden Gate assembly, with the following vectors: the Gibson-assembled vectors pJJB308-CasRx-(gRNA) and pJJB296-CasRx, and intermediate module vector pMOD_C0000 (Addgene; plasmid #91081) and transformation backbone vector pTRANS_220d (Addgene; plasmid #91114). For the assembly of the vector without a gRNA (pCasRx-EMPTY), the modified pJJB308-CasRx-backbone plasmid was used in the Golden Gate assembly reaction. The reaction was set up as described in the paper by Čermák and colleagues (Čermák et al., 2017).

The gRNAs for pTRV2 (SPDK3876 (TRV2-pPEBV-MCS), Addgene plasmid #149275) were designed to contain the CasRx-scaffold sequence (5’-AACCCCTACCAACTGGTCGGGGTTTGAAACG-3’) at the 5’-end, and gRNA sequence with poly-T-tail [5’-(gRNA sequence)TTTTTTTTT-3’] at the 3’-end, as well as XbaI and XhoI overhangs on the 5’- and 3’-end respectively (**Supplementary Table 1**). The targets which were to be cloned into TRV were ordered and synthesized as ssDNA oligos from IDT (Integrated DNA Technologies, USA). The oligos were phosphorylated using T4 Polynucleotide Kinase (Promega, USA), following the manufacturer’s protocol. The phosphorylated oligos were annealed as previously described. The TRV2 vector was digested with XbaI and XhoI and ligated with the phosphorylated and annealed gRNAs.

### 2.3 *N. benthamiana* transformation

Overnight-grown cultures of *A. tumefaciens* strain EHA105 containing the construct pCasRx-EMPTY were centrifuged and suspended in sterile MS3 broth (4.4g/L Murashige & Skoog (Murashige and Skoog, 1962) (MS) medium, 30g/L sucrose, 100µg/mL acetosyringone, pH 5.8) to an optical density (OD)_600_ of 0.8-1. Leaves of wild-type *N. benthamiana* grown *in vitro* were dissected into approximately 50 leaf disc explants of 1cm^2^ each, and used for *Agrobacterium*-mediated stable transformation, as described by Clemente (Clemente, 2006). Shoot organogenesis was induced on leaves cultured on selective medium (4.4g/L MS, 30g/L sucrose, 3.3g/L PhytoAgar, 1mL 6-Benzylaminopurine (BAP) (1µM), pH 5.8), incubated at 25°C with a 16hr/8hr light:dark, and explants on fresh selective medium every 14 days. Regenerated shoots were excised from calli and placed on hormone-free rooting medium (half-strength MS, 15g/L sucrose, 3.3g/L PhytoAgar (Duchefa Biochemie, NL), pH 5.8) containing 100µg/mL kanamycin and 400µg/mL carbenicillin, and maintained in Magenta™ pots.

### 2.4 Confirmation of transgenic events

Leaves were harvested from *in vitro*-grown plantlets, and the standard cetyltrimethylammonium bromide (CTAB) method (Murray and Thompson, 1980) was used for genomic DNA extraction. Putative transgenic plants were screened with an end-point PCR, using the primers CaMV-35S_F2/CasRx-screening_R1 (**Supplementary Table 2**). Positive lines were propagated until the desired number of plants for that line were achieved.

### 2.5 Agro-infiltration of *N. benthamiana* plants

**Figure 1:**
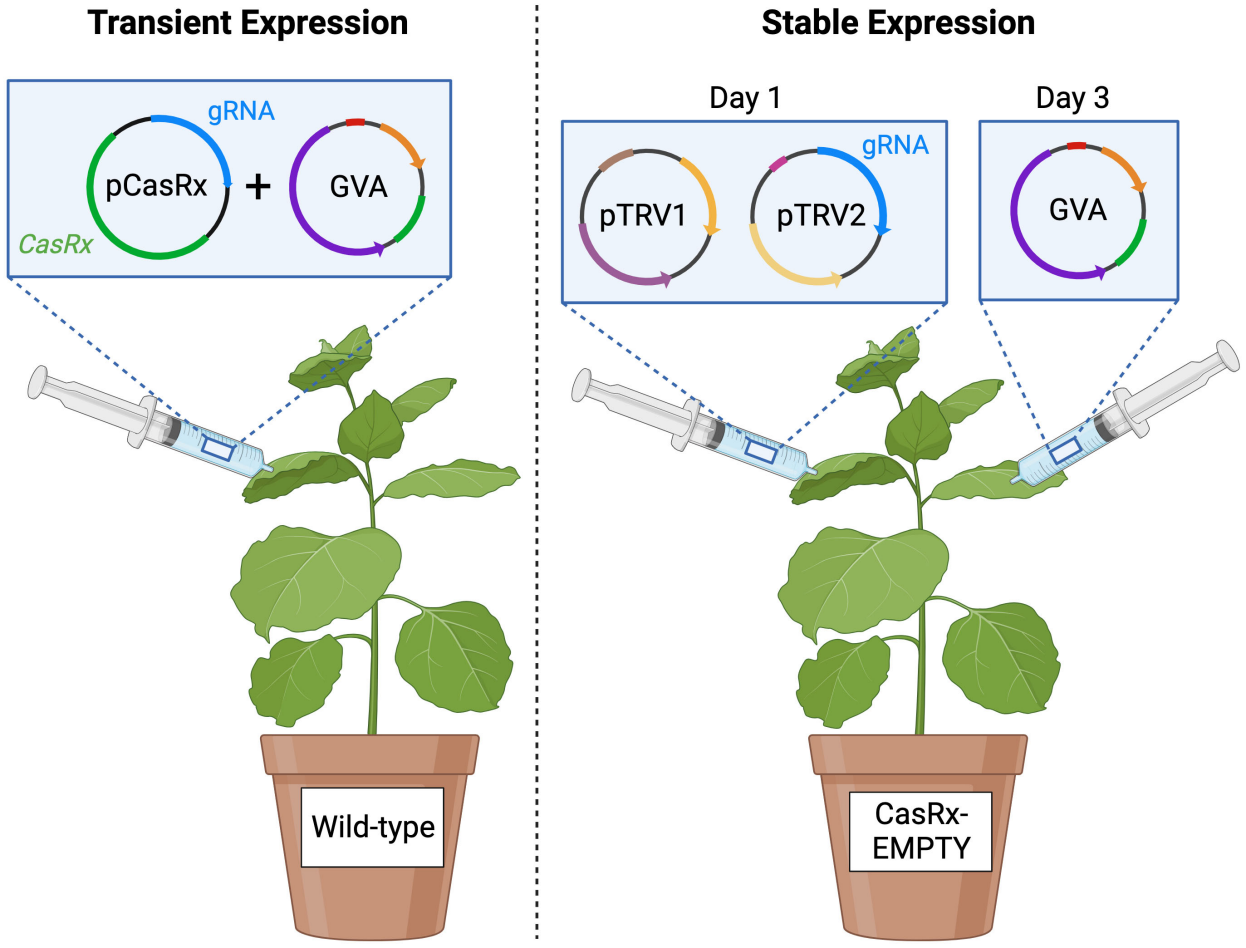
Schematic representing the transient expression experiment and the stable expression experiment. *Agrobacterium* mixtures containing the binary pCasRx construct harbouring *CasRx* and a desired gRNA (or without gRNA for the control), and the GVA infectious clone, were mixed in a 1:1 ratio and infiltrated into wild-type *N. benthamiana* (left). *Agrobacterium* mixture of pTRV1 and pTRV2 harbouring a desired gRNA were mixed in a 1:1 ratio and infiltrated into *N. benthamiana* stably-expressing CasRx (CasRx-EMPTY plants), followed by the GVA infectious clone 48 hours later. Created with BioRender.com.

*A. tumefaciens* strain EHA105 harbouring the binary pCasRx vectors containing the respective CP gRNAs, named pCasRx : CP_T1, pCasRx : CP_T2, pCasRx : CP_T3 were grown overnight with 50µg/mL kanamycin and 30µg/mL rifampicin. Cultures were centrifuged at 1000 RPM for 15 minutes at room temperature, and resuspended in infiltration buffer (10mM MES [pH 5.6], 10mM MgCl_2_, 200µM acetosyringone) to a final OD_600_ of 0.8-1.0. The resuspended cultures were incubated in the dark at ambient room temperature for 2 hours. Mixed cultures at a 1:1 ratio of the pCasRx constructs and the GVA infectious clone were infiltrated in three fully expanded four-week-old wild-type *N. benthamiana* leaves grown under long-day conditions (16-hour light, 8-hour dark at 25°C), using a needleless 2mL syringe, for transient-expression experiments (**Figure 1** left).

Cultures of *A. tumefaciens* strain GV3101 containing the unmodified pTRV1 (pYL192, Addgene plasmid #148968) vector, and the modified pTRV2 vectors harbouring the CP gRNAs (pTRV2:CP_T1, pTRV2:CP_T2, pTRV2:CP_T3) were grown overnight, containing 50µg/mL kanamycin, 10µg/mL rifampicin and 30µg/mL gentamicin. Cultures were centrifuged at 1000 RPM for 15 minutes at room temperature, and resuspended in infiltration buffer to OD_600_ 1. The culture of the GVA clone (OD_600 =_ 0.05) was mixed with the TRV mixture (containing either pTRV2:CP_T1, pTRV2:CP_T2, pTRV2:CP_T3) and infiltrated into three leaves of CasRx-EMPTY plants, for single-gRNA experiments. For multi-gRNA experiments, the desired combinations of pTRV2:CP vectors were mixed in an equal ratio, which was then subsequently mixed in a 1:1 ratio with pTRV-RNA1. The TRV mixture was infiltrated two days before the GVA clone. The mixtures were infiltrated as previously described, into either wild-type *N. benthamiana* or CasRx-EMPTY plants (**Figure 1**).

### 2.6 RNA extraction and cDNA synthesis

Infiltrated wild-type *N. benthamiana* leaf material was harvested 5-days post-infiltration (dpi) for transient experiments, or 5-dpi from GVA infiltration for stable experiments (7-dpi from TRV mixture infiltration), and three infiltrated leaves per plant were pooled together and subsequently frozen in liquid nitrogen. Total RNA was extracted from 100mg aliquots using the Spectrum™ Plant Total RNA Kit (Sigma, USA) following the manufacturer’s instructions. First-strand complementary DNA (cDNA) synthesis was performed using 1µg of DNase-treated total RNA with Random Primers (Promega, USA) and Maxima Reverse Transcriptase (Thermo Fisher Scientific, USA), following the manufacturer’s instructions. cDNA synthesis was confirmed with an end-point PCR by amplifying the housekeeping gene *actin*.

### 2.7 Reverse-transcription quantitative-PCR expression analysis

A QuantStudio™ 3 Real-Time PCR System (Applied Biosystems, USA), along with the reporter PowerUp™ SYBR™ Green Master Mix (Applied Biosystems, USA), were used for expression analysis. The housekeeping gene adenine phosphoribosyltransferase like (*APR*) (Liu et al., 2012) was chosen as the internal control (**Supplementary Table 3**), and the desired RT-qPCR primer sets for gene expression analysis are listed in **Supplementary Table 3**. The comparative C_T_ (ΔΔ C_T_) method was selected and the cycle parameters were set for Standard cycling mode (Primer T_m_≥ 60°C): 50°C/2min + 95°C/2min + 40x (95°C/15sec + 60°C/1min), and the melt curve parameters were set as the default settings. The 2^-ΔΔCT^ method was used to calculate the gene expression levels, relative to the reference sample, in the online Design and Analysis app (Thermo Fisher Scientific, USA). The error bars serve as an indication between the RQ minimum and RQ maximum for the sample. A confidence interval of 95% was used.

### 2.8 Statistical analysis

The relative gene expression of GVA was analyzed statistically using a two-tailed unpaired Student’s t-test. Data represents the mean ± standard error of the mean (SEM), and the significance determination was set at p ≤ 0.05. The Pearson correlation coefficient (r) was used to calculate the correlation between the relative gene expression of the respective gRNA compared to the expression of the GVA *CP* gene. GraphPad Prism (version 9.1.1, GraphPad Software, Boston, Massachusetts USA) software was used to compute the analyses.

## 3 Results

### 3.1 Stable transformation of *N. benthamiana* plants with pCasRx constructs

**Figure 2:**
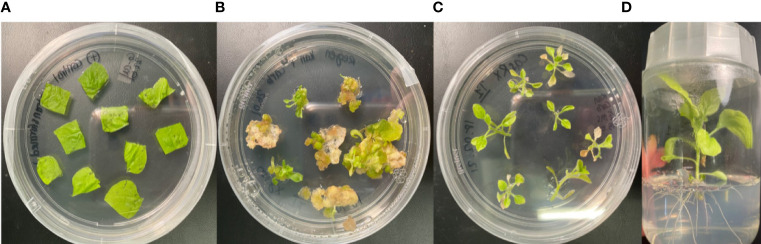
*Agrobacterium*-mediated stable transformation and regeneration of wild-type *N. benthamiana* leaf disc organogenesis process. **(A)** Leaf discs on co-cultivation medium immediately after incubation with the *Agrobacterium* harbouring the respective constructs. **(B)** Callus stage with shoots beginning to form on selective medium. **(C)** Isolated shoots on rooting medium containing selection. **(D)** Fully regenerated putative transgenic plantlet on selective medium.

*N. benthamiana* plants stably expressing CasRx are a useful tool for the testing of different gRNAs. The transformation process and results are illustrated in **Figure 2**: The leaf discs used for co-cultivation (**Figure 2A**); developed callus and shoots on a selective medium (**Figure 2B**); the shoots were isolated and cultured on a rooting medium (**Figure 2C**); and rooted plants were maintained on a selection medium (**Figure 2D**). Regenerated plantlets were screened to identify positive lines. Two positive lines from the pCasRx-EMPTY construct stable transformation were confirmed and propagated further.

### 3.2 Coat protein gRNA design

**Figure 3:**
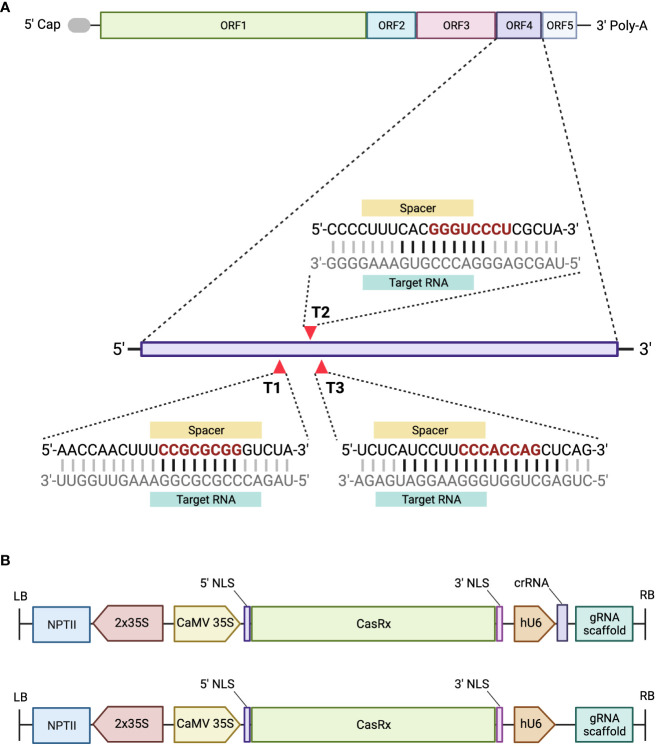
A schematic representing the GVA genome organization and the designed gRNAs, and the assembled constructs. **(A)** GVA genome organization, ORF1 encodes replication-related proteins; ORF2 encodes a protein with an unknown function; ORF3 encodes the movement protein (MP); ORF4 encodes the CP, and ORF5 encodes a silencing-suppressor protein. Three gRNAs were designed to target ORF4 of GVA. The seed region of the gRNA sequence is represented in red. The black horizontal lines between the gRNA and target RNA represent the nucleotides complementing single-stand RNA. **(B)** Schematic representation of the T-DNA region of the pCasRx-gRNA or pCasRx-EMPTY constructs, assembled using Golden Gate cloning. The neomycin phosphotransferase type II gene (*NPTII*) is driven by 2x35S promoters. The cauliflower mosaic virus (CaMV) 35S promoter drives the *CasRx* expression, while the human U6 (hU6) promoter is responsible for expressing the respective gRNA and gRNA scaffold. NLS, nuclear localization signal; LB, left border; RB, right border. Created with BioRender.com.

Open reading frame four (ORF4) of pBINSN_GVA118_NbPD encodes the *CP* gene, and was chosen as the target for CRISPR/CasRx virus interference, as previous literature had observed decreased viral accumulation when the CP was targeted (Aman et al., 2018a; Aman et al., 2018b). Three gRNAs were designed against the *CP* gene of GVA, in a region which revealed increased accessibility (**Figure 3A**). The cas13design software output ranks the gRNA in number and quartile, where quartile 4 (Q4) are the best scored and quartile 1 (Q1) are the worst scored gRNAs. Accessibility of the seed region (position 11-18 nt) and single-stranded regions were emphasized during the gRNA selection process (Bandaru et al., 2020), as well as the folding of the gRNA with the scaffold sequence (**Supplementary Figure 2**). The CP gRNAs selected were: Target 1 (T1) (rank 90, Q3), Target 2 (T2) (rank 3, Q4), and Target 3 (T3) (rank 164, Q3) (**Figure 3A**). The sequences of each target’s seed region were searched against the *N. benthamiana* transcriptome to identify possible off-targets: T1 contained two off-targets which complemented the seed region, but never the entire gRNA; T2 contained the most off-target possibilities (15) for the seed region, but never the entire gRNA sequence; and T3 contained no off-targets complementing the seed region, nor the rest of the gRNA.

### 3.3 Transient expression does not lead to consistent GVA interference

**Figure 4:**
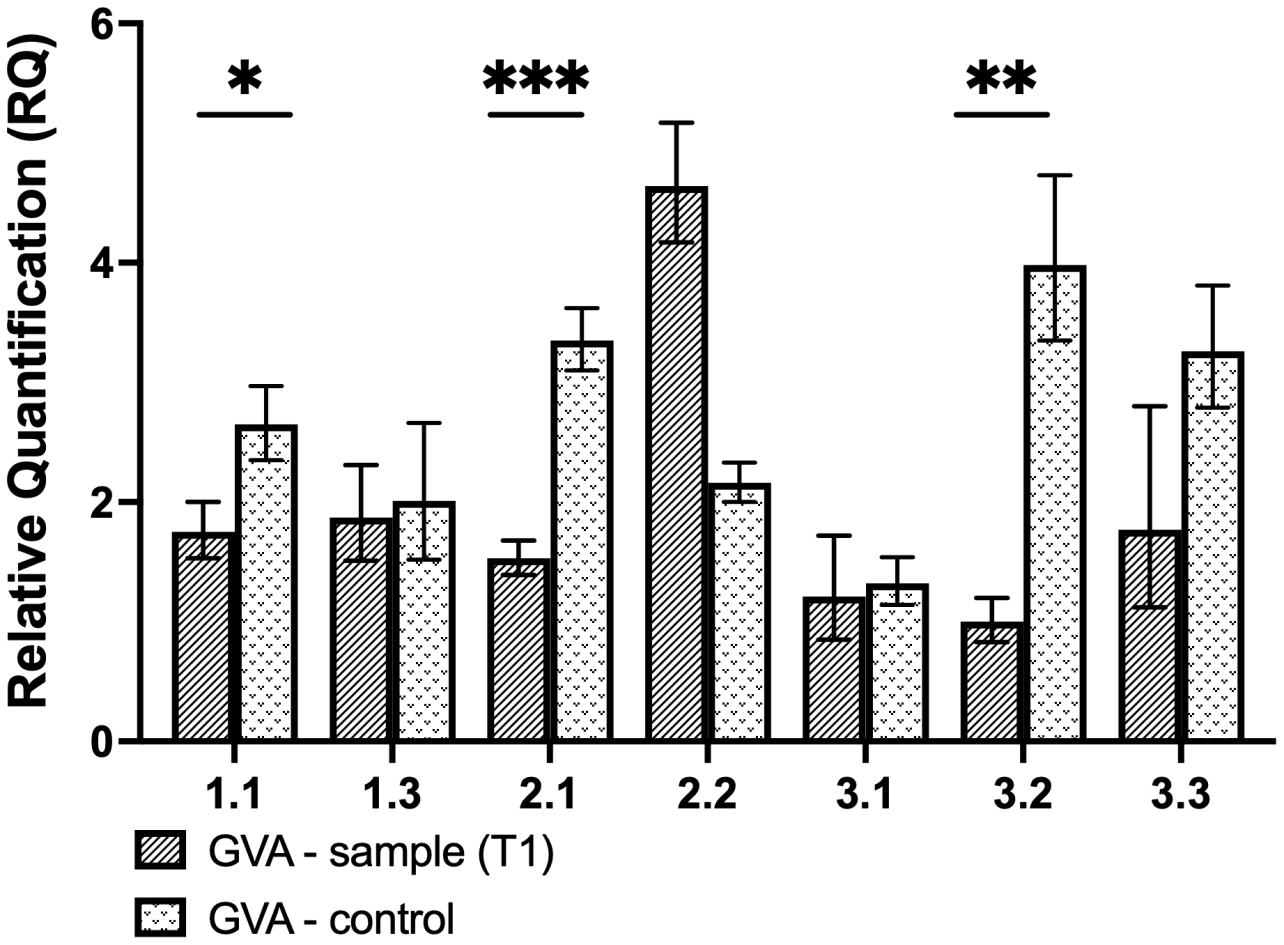
Relative quantification of GVA for transient experiment analysis. The control was infiltrated with the pCasRx-ns vector, while the test sample was infiltrated with pCasRx : CP-T1. The error bars represent the minimum and maximum values of n=3 technical replicates. Statistical analysis performed using a two-tailed unpaired Student’s t-test; with the difference being significant at p ≤ 0.05 with respect to the control group. * p=0.0163, ** p=0.0019, *** p=0.0004.

As a preliminary analysis, transient assays were performed to assess the efficacy of the individual CP gRNAs to induce GVA interference. The expression of the *CasRx* gene was driven by the CaMV 35S promoter, while the gRNA was driven by the hU6 promoter (**Figure 3B**). Wild-type *N. benthamiana* plants were co-infiltrated with the GVA clone and the different binary vectors pCasRx : CP_T1, pCasRx : CP_T2 and pCasRx : CP_T3 (right side of the leaf). As a control, co-infiltrations of the binary vector containing a non-specific (ns) gRNA (pCasRx-ns), along with the GVA clone, were performed (left side of the leaf). For gRNA CP-T1, virus interference was observed in three samples (**Figure 4**). Indeed, GVA quantification for sample 1.1, 2.1, and 3.2 were ~2-fold, ~4-fold, and ~1.5-fold reduced, respectively. However, when CP-T2 or CP-T3 were used, no samples showed a decrease in GVA, when compared to the control samples infiltrated with a ns gRNA (pCasRx-ns).

### 3.4 CRISPR/CasRx interference of GVA with a single gRNA targeting the coat protein

**Figure 5:**
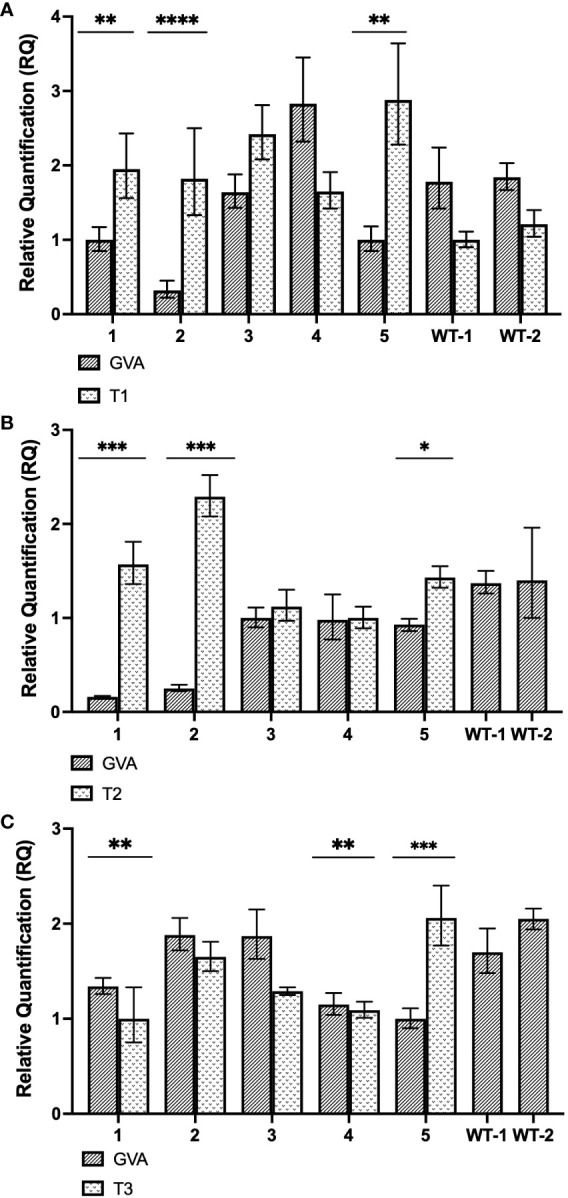
Gene expression analysis of stably-transformed *N. benthamiana* (CasRx-EMPTY plants). **(A)** Relative fold expression of GVA and T1, subsequent to the GVA clone and pTRV2:CP-T1 co-infiltration into CasRx-EMPTY plants. ** p<0.0027, **** p<0.0001. **(B)** Relative fold expression of GVA and T2, subsequent to the GVA clone and pTRV2:CP-T2 co-infiltration in CasRx-EMPTY plants. * p=0.0038, *** p<0.0005. Correlation coefficient (r) is –0.7738 (p=0.024). **(C)** Relative fold expression of GVA and T3, subsequent to GVA and pTRV2:CP-T3 co-infiltration into CasRx-EMPTY plants. The error bars represent the minimum and maximum values of n=3 technical replicates. Statistical analysis performed using a two-tailed unpaired Student’s t-test; with the difference being significant at p ≤ 0.05 with respect to the control group. Correlation analysis performed using the Pearson correlation coefficient (r); with the correlation being significant at p ≤ 0.05. ** p<0.0095, *** p=0.0007.

CasRx-expressing plants (CasRx-EMPTY) were used to determine the capability of the CRISPR/CasRx system to confer virus interference against GVA, when gRNAs targeting the CP were delivered with TRV. Subsequent to the co-infiltration of the GVA clone and pTRV2 vector harbouring the respective gRNA, the relative fold expression of GVA and the gRNA were analyzed 5-dpi (**Figure 5A**). As the control, two wild-type *N. benthamiana* plants were infiltrated with GVA and pTRV2:CP-T1.

As shown in **Figure 5A**, after infiltration with pTRV2:CP-T1, GVA interference was observed in sample 1, 2 and 5, as there was a significant difference between GVA quantification in the sample compared to the two wild-type *N. benthamiana* control plants (WT1, WT2). Of the three samples, sample 2 indicated the highest GVA reduction when compared to the control samples (~5-fold reduction, p<0.0001). In **Figure 5B**, three samples (sample 1, 2 and 5) demonstrated GVA interference when pTRV2:CP-T2 was infiltrated in CasRx-EMPTY, compared to the WT controls. The highest GVA interference was an ~8-fold reduction, observed in sample 1 and 2 (p< 0.0005), when compared to WT1. The relative T2 gRNA expression was negatively correlated with virus accumulation, at the P ≤ 0.05 level. The Pearson correlation coefficient was –0.7738. Lastly, when pTRV2:CP-T3 was infiltrated, samples 1, 4 and 5 (**Figure 5C**) had significantly lower GVA quantification compared to WT1 and WT2, with sample 5 demonstrating a ~1.7-fold reduction in GVA (p=0.0007).

### 3.5 GVA inhibition with multi-gRNA CRISPR/CasRx system but not guide-induced gene silencing

**Figure 6:**
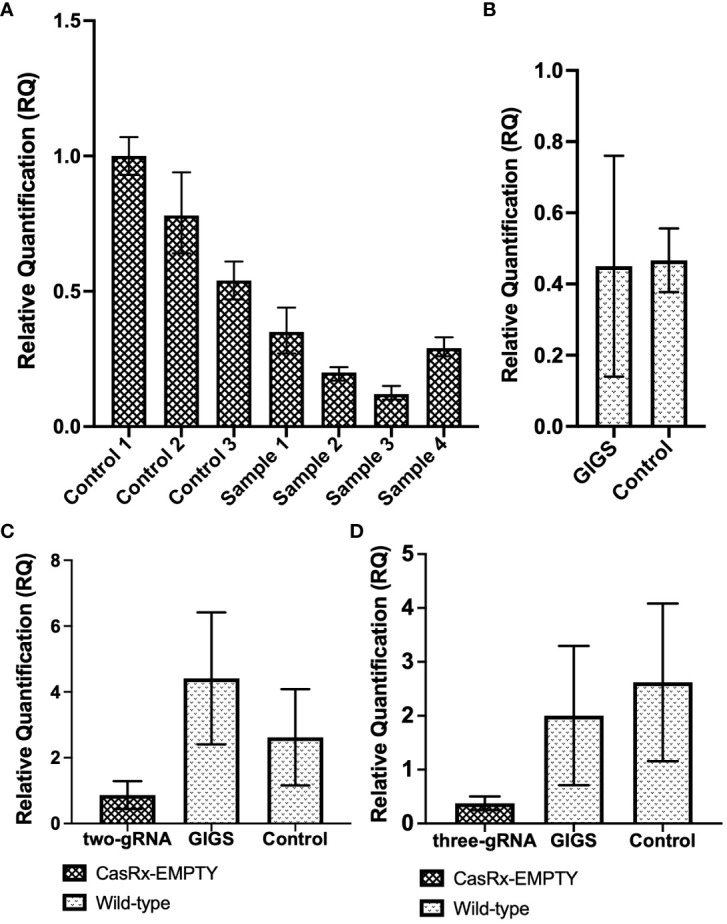
GVA interference by CRISPR/CasRx compared to guide-induced gene silencing. **(A)** Relative fold expression of GVA in CasRx-EMPTY plants, normalized to the reference sample ‘control 1’. Samples ‘control 1 to 3’ infiltrated with TRV-EMPTY, and GVA two-dpi. Samples labelled ‘sample 1 to 4’ infiltrated with the multi-gRNA TRV mixture, and GVA two-dpi. The error bars represent the minimum and maximum values of n=3 technical replicates. **(B)** Relative quantification of GVA expression in wild-type *N. benthamiana*. Data represents the mean relative gene expression, ± SEM of the biological replicates (n=3). **(C)** Relative quantification of GVA expression of the ‘two-gRNA’ samples in CasRx-EMPTY and wild-type plants. The control sample was infiltrated with TRV-EMPTY, and GVA two-dpi, in wild-type *N. benthamiana*. Data represents the mean relative gene expression, ± SEM of the n=3 samples (controls), n=3 samples (GIGS) and n=5 samples (two-gRNA). **(D)** Relative quantification of GVA expression of the ‘three-gRNA’ samples in CasRx-EMPTY and wild-type plants. The control sample was infiltrated with TRV-EMPTY, and GVA two-dpi, in wild-type *N. benthamiana*. Data represents the mean relative gene expression, ± SEM of the n=3 samples (controls), n=3 samples (GIGS) and n=5 samples (three-gRNA). Statistical analysis performed using a two-tailed unpaired Student’s t-test; with the difference being significant at p ≤ 0.05 with respect to the control.

To analyze virus interference with a multi-guide system, as well as GIGS, the GVA infectious clone was infiltrated into CasRx-EMPTY plants, as well as into wild-type *N. benthamiana* plants, two days after the TRV : CP guides were infiltrated. **Figure 6** shows the relative quantification of GVA (see GVA_qPCR CP_F1/GVA_qPCR CP_R1 primers in **Supplementary Table 3**) in both CasRx-EMPTY plants and wild-type plants.

When all three CP gRNAs were infiltrated (**Figure 6A**), all samples showed a reduction in GVA accumulation, with ‘sample 3’ having the lowest GVA accumulation, indeed a >3.5-fold reduction compared to ‘control 1 to 3’ (infiltrated with TRV-EMPTY and GVA) was observed. When the GIGS samples were analyzed for GVA reduction (**Figure 6B**), no clear difference in GVA accumulation was observed in the ‘GIGS’ sample when compared to the control samples which did not receive gRNAs. (**Figure 6B**).

In the next experiment, a two-guide system was compared to a three-guide system. In **Figure 6C**, the ‘two-gRNA’ CasRx-EMPTY plants group presented a ~3-fold reduction in GVA, when compared to the control group, although the reduction was not significant, while GIGS samples showed no reduction in GVA quantification, compared to the controls. When CasRx-EMPTY plants were infiltrated with all three CP gRNAs (three-gRNA), followed by GVA two days later, there was a ~5-fold reduction in GVA accumulation, when compared to the control group (**Figure 6D**). The GIGS group also showed lower GVA quantification (~>1-fold reduction), compared to the control group. Although a reduction in GVA for both GIGS and ‘three-gRNA’ samples was observed, the reduction was not significant when p ≤ 0.05.

### 3.6 Highly effective gRNA delivery with TRV

**Figure 7:**
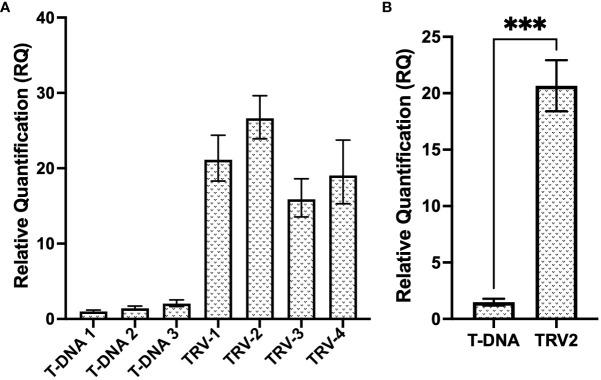
Relative expression levels of the T2 gRNA from a regular T-DNA binary vector and a TRV viral vector. **(A)** Relative fold expression of T2 gRNA expressed under the hU6 promoter, from the T-DNA of the pCasRx : CP-T2 vector, compared to the expression of T2 gRNA under the PEBV promoter, from the pTRV2:CP-T2 vector. The error bars represent the minimum and maximum values of n=3 technical replicates. **(B)** Relative quantification of T2 gRNA expression of the T-DNA samples and TRV2 samples grouped biologically. Data represents the mean relative gene expression, ± SEM of the three and four biological replicates, respectively (n=3, n=4). Statistical analysis performed using a two-tailed unpaired Student’s t-test; with the difference being significant at p ≤ 0.05 with respect to the TRV2 group. *** p=0.0008.

In order to assess the efficiency of a TRV vector at delivering and expressing the CP gRNA transiently and systemically, RT-qPCR analysis (see primers CasRx_gRNA-scaf_F/CP_T2_qPCR_R in **Supplementary Table 3**), compared the relative expression of the T2 gRNA, expressed either under the hU6 promoter from the binary pCasRx vector, or under the PEBV promoter, from the TRV2 vector (**Figure 7A**). The relative fold increase in gRNA expression from the TRV2 vector, was >15-fold, when compared to ‘T-DNA 1’. When the samples were grouped, a ~13-fold increase was observed when gRNA T2 was expressed from TRV2, compared to regular T-DNA (**Figure 7B**).

## 4 Discussion

Plant viruses are responsible for causing agricultural devastation and great economic losses worldwide. The majority of these viruses possess RNA genomes, and in an attempt to control plant viruses, researchers have utilized the RNA-targeting CRISPR/Cas13 system to confer virus resistance in plants. While this method provides the means of controlling viruses, the efficient delivery of CRISPR components in plant cells has proven to be a bottleneck to scientists (Laforest and Nadakuduti, 2022). To overcome this, some viruses have been harnessed and manipulated to deliver genome editing components *in planta*. Specifically, the bipartite virus, TRV, has been harnessed for the efficient expression of gRNAs in numerous studies (Aman et al., 2018b; Mahas et al., 2019). In the current study, the CRISPR/CasRx system was used for targeting of the RNA grapevine virus, GVA, by employing the conventional T-DNA expression system and TRV-gRNA expression system in transgenic *N. benthamiana*.

Three new gRNAs were designed which targeted the *CP* gene of GVA, a common gene targeted in literature (Aman et al., 2018a; Aman et al., 2018b). The activity of the CRISPR/Cas13 system is dependent on the target RNA secondary structure (Abudayyeh et al., 2017; Mahas et al., 2019; Bandaru et al., 2020; Wessels et al., 2020), and a study found that the most effective gRNAs with the strongest interference were clustered together (Abudayyeh et al., 2016). Importantly, the central seed region of the gRNA should target single-strand RNA regions for efficient RNA interference (Bandaru et al., 2020). Therefore, the CP gRNAs for this experiment were clustered together within a more accessible region of ORF4, and the accessibility of the central seed region was emphasized during the selection process.

Transient assays to assess the virus interference efficiencies of the newly-designed gRNAs were performed as preliminary tests. Of the three gRNAs assayed transiently, T1 was the only gRNA that demonstrated GVA interference in three samples (**Figure 4**). One study by the Aman group found that when transient assays were performed, a 50% reduction in virus accumulation was observed (Aman et al., 2018b). It is important to note that the binary vector used in the Aman group study harbored the *Cas13a* gene, while the gRNAs targeting TuMV-GFP were expressed by the TRV delivery system, and successful TuMV-GFP targeting was observed 7-dpi in systemic leaves. A clear difference between the Aman group study and the current study, was that the gRNA in the present study was expressed from a binary vector under the hU6 promoter, and not from TRV under the PEBV promoter (Aman et al., 2018b). Similarly, another study found that when the expression of the CasRx gRNA was driven by the AtU6 promoter, no efficient RNA targeting was achieved, and when the promoter was exchanged for the cestrum yellow leaf curling virus (CmYLCV) promoter, RNA interference was remarkably increased (Yu et al., 2022). To understand why only T1 resulted in GVA interference in the transient assays, gene expression analysis was performed to assess the gRNA expression under the hU6 promoter, and compare it to the TRV-based gRNA expression under the PEBV promoter (**Figure 7**). The analysis clearly demonstrated the difference in expression levels (~13-fold) and confirmed that gRNA delivery and expression from the TRV-PEBV system is more efficient. Although it is not clear why targeting efficiency was lower in transient assays in the present study, this could be attributed to lower gRNA expression (Yu et al., 2022).

By harnessing the constitutively expressed *CasRx* gene, and delivering the gRNAs transiently with TRV, multiple gRNAs could be assayed in parallel. For CRISPR/CasRx interference, the CP-targeting gRNAs were delivered into transgenic CasRx-EMPTY plants. **Figure 5A** depicted three samples which show reduced GVA quantification. In this experiment, the wild-type plants infiltrated with T1 gRNA were used as a control. Importantly, prior to this experiment, there had not been sufficient literature regarding the occurrence of GIGS (Sharma et al., 2022). GIGS functions in the absence of Cas13, where the gRNA alone can elicit reductions in viral RNA (Sharma et al., 2022). Considering the possibility of GIGS, our control may not have been accurate, as GIGS may have influenced the virus accumulation. Ultimately, the results for T1 did not show much consistency. The results of T2 presented more consistent GVA quantifications, and virus interference seemed to have taken place (**Figure 5B**). The correlation which existed between the gRNA expression levels and the GVA quantification (-0.7738) indicated that GVA quantification was dependent on gRNA expression, which was corroborated by previous literature (Yu et al., 2022).

The varying efficiencies of gRNAs has been observed previously in literature, and may be explained by the formation of secondary structures within the RNA target region, or the presence of RNA binding proteins which can affect the accessibility of the Cas13 nuclease with the target RNA (Abudayyeh et al., 2016; Abudayyeh et al., 2017; Smargon et al., 2017; Aman et al., 2018b; Liu et al., 2023). Therefore, the target RNA accessibility is an important factor which governs the activity of Cas13 (Aman et al., 2018b). Thus, the difference in efficiency of T1, T2 and T3 may be as a result of the accessibility of the target RNA. A study found that the efficiency of the gRNA can be diminished if the seed region of the target RNA is not entirely accessible (Wessels et al., 2020). In contrast to this, all eight nts of the seed region of T1 and T3 are supposedly accessible, while only six nts of the T2 seed region are accessible. The study by Wessels et al. (2020) determined that gRNAs ranked within the top two quartiles (Q4 and Q3) resulted in more efficient RNA interference than gRNAs ranked in the bottom two quartiles (Q2 and Q1). Interestingly, T2 was ranked the 3^rd^ gRNA (Q4), while T1 was ranked 90^th^ (Q3), and T3 was ranked 164^th^ (Q3), which supports the finding in this study, that T2 seemed to result in the highest virus interference. Another important factor which can determine targeting efficiency is the secondary structure formation within the CasRx-gRNA-scaffold, as this determines binding of the CasRx:gRNA complex and target localization (Zhang et al., 2018). Of the three CP gRNAs, T2 showed the most precise folding with the scaffold sequence (Zhang et al., 2018; Mahas et al., 2019), while T3 had the least precise folding (**Supplementary Figure 2**), also supporting the finding that T2 was the more efficient gRNA.

Literature has shown that a multi-guide system resulted in more robust RNA interference, compared to single gRNA systems (Sharma et al., 2022). Thus, a multi-guide system was assayed and compared in CasRx-EMPTY plants and wild-type *N. benthamiana* plants. GVA reductions seen in CasRx-EMPTY plants but not in wild-type plants were corroborated by literature, which described that RNA interference was most efficient when the Cas13 nuclease was present (Sharma et al., 2022). The lack of interference by GIGS may be explained by the fact that the CP gRNAs designed in this study were only 23 nts in length. Efficient RNA interference by GIGS rises with an increased gRNA length, and guides below 20 nts in length did not result in GIGS (Sharma et al., 2022). For GIGS to result in viral interference, the gRNA length should be between 24 to 28 nts in length (Sharma et al., 2022).

The constructs pTRV1 and pTRV2-EMPTY were infiltrated together as a negative control. A two- and three-guide approach was utilized and compared in CasRx-EMPTY plants and wild-type *N. benthamiana*. CasRx-EMPTY samples in **Figure 6C and D** both showed lower GVA accumulation when compared to the GIGS and control samples, although the reduction was not significant. This result was supported by a study which found that a multi-guide approach, in the presence of Cas13, results in increased RNA interference (Sharma et al., 2022). When the two-guide system was compared to the three-guide system, the latter system seemed to cause more efficient virus interference. Once again, GIGS did not seem to result in much GVA interference, most likely due to the length of the gRNAs (Sharma et al., 2022). It is important to note that in the present study, the CasRx localization signal was a nuclear localization signal (NLS). However, one study found that the CasRx nuclease fused to a nuclear export signal (NES) resulted in the highest RNA interference when targeting RNA viruses, while CasRx-NLS also demonstrated RNA virus interference, albeit at a lower efficiency (Mahas et al., 2019). This observation was most likely explained by the simultaneous localization of CasRx and the RNA virus, which replicates in the cytoplasm (Mahas et al., 2019). Additionally, the significant variation observed among samples may be due to the Agro-infiltration process. A study found high within-leaf variation and between-leaf variation existed when quantitative assays were performed in *N. benthamiana* (Bashandy et al., 2015).

In conclusion, when targeting the *CP* gene of GVA, virus interference was observed, specifically T2, when *CasRx*-expressing plants were infiltrated with TRV-delivered gRNAs. The multi-guide infiltrations proved to result in lower GVA accumulation, specifically when all three CP gRNAs were co-infiltrated. Additionally, it was found that GIGS did not result in RNA interference in this case. Future considerations to improve GVA targeting might include using the CasRx-NES variant, as replication of GVA occurs in the cytoplasm, synchronizing the localization of CasRx and viral replication. Although TRV-gRNA expression was relatively efficient in this study, the use of viral RNA silencing suppressors co-expressed with TRV can further improve expression of the system (Chiong et al., 2021). Importantly, the design of efficient gRNAs is essential to achieve RNA targeting, taking into consideration the use of multiple gRNAs and secondary structure formation. CRISPR/Cas systems offer unprecedented opportunities for gene engineering, and continuous advancements in the field of CRISPR editing are expanding the possibilities available to scientists. The agricultural sector is afflicted by plant viruses and the diseases they cause, and thus sustainable solutions are necessary to control these viruses. This study achieved moderate virus interference with an RNA-targeting CRISPR/CasRx system in a model plant and contributes to finding a solution to control plant viruses that plague economically important crops, such as grapevine.

## Data availability statement

The original contributions presented in the study are included in the article/**Supplementary Material**. Further inquiries can be directed to the corresponding author.

## Author contributions

KPS: Data curation, Investigation, Methodology, Writing – original draft. JTB: Conceptualization, Funding acquisition, Resources, Supervision, Writing – review & editing. MC: Conceptualization, Funding acquisition, Methodology, Project administration, Supervision, Writing – review & editing.
