# Supplementary material for: CRISPR-based resistance to grapevine virus A
Source: Front Plant Sci. 2023 Dec 4;14:1296251. doi: 10.3389/fpls.2023.1296251 (PMC10725905; doi:10.3389/fpls.2023.1296251)
Supplement: Supplementary file 1 [file DataSheet_1.docx]

Supplementary Material

|  |  |
| --- | --- |

**Supplementary Figure 1.** Intermediate vector maps of plasmids modified by Gibson assembly. Vector pJJB296 (left) contains the *CasRx* gene. Vector pJJB308 (right) contains the CasRx-gRNA backbone.

| (**A**) |  |  |
| --- | --- | --- |
| (**B**) |  | **** |
| (**C)** |  |  |

**Supplementary Figure 2.** Predicted RNA folding of coat protein (CP) guide RNA (gRNA) and CasRx‑gRNA-scaffold sequences, with RNAfold (left) and mFold (right) software. (**A**) CP gRNA Target 1 and scaffold folding. (**B**) CP gRNA Target 2 and scaffold folding. (**C**) CP gRNA Target 3 and scaffold folding. The RNAfold structure is colored by base-pairing probabilities, for unpaired regions the colour denotes the probability of being unpaired.

**Supplementary Table 1.** gRNA oligonucleotides used for GVA coat protein targeting.

| **Oligo name** | **Sequence (5’-3’)** |
| --- | --- |
| GVA_CP_T1_F | AAACGAACCAACTTTCCGCGCGGGTCTA |
| GVA_CP_T1_R | AAAATAGACCCGCGCGGAAAGTTGGTTC |
| GVA_CP_T2_F | AAACGTCTCATCCTTCCCACCAGCTCAG |
| GVA_CP_T2_R | AAAAACTGAGCTGGTGGGAAGGATGAGA |
| GVA_CP_T3_F | AAACGCCCCTTTCACGGGTCCCTCGCTA |
| GVA_CP_T3_R | AAAATAGCGAGGGACCCGTGAAAGGGGC |
| CP_T1_gRNA_F | ctagaacccctaccaactggtcggggtttgaaacgAACCAACTTTCCGCGCGGGTCTATTTTTTTTT |
| CP_T1_gRNA_R | tcgaAAAAAAAAATAGACCCGCGCGGAAAGTTGGTTcgtttcaaaccccgaccagttggtaggggtt |
| CP_T2_gRNA_F | ctagaacccctaccaactggtcggggtttgaaacgTCTCATCCTTCCCACCAGCTCAGTTTTTTTTT |
| CP_T2_gRNA_R | tcgaAAAAAAAAACTGAGCTGGTGGGAAGGATGAGAcgtttcaaaccccgaccagttggtaggggtt |
| CP_T3_gRNA_F | ctagaacccctaccaactggtcggggtttgaaacgCCCCTTTCACGGGTCCCTCGCTATTTTTTTTT |
| CP_T3_gRNA_R | tcgaAAAAAAAAATAGCGAGGGACCCGTGAAAGGGGcgtttcaaaccccgaccagttggtaggggtt |

**Supplementary Table 2.** Primers used for cloning and RT-qPCR analysis.

| **Primer name** | **Sequence 5’-3’** | **Tm (°C)** | **Amplicon (bp)** |
| --- | --- | --- | --- |
| gRNA_backbone_fwd | aagagcgcccacctgccaggggacGAGGGCCTATTTCCCATG | 55.6* | 302 |
| gRNA_backbone_rev | gcttttcatcacctgcgtcaccggaaaAAAAAGGTCTTCTCGAAGAC | 49.7* |  |
| CasRx_fwd | acgggggactcccgacatggCCCAAGAAGAAGAGAAAG | 46.2* | 3000 |
| CasRx_rev | aacacaaacttaagcacacaCACCTTCCTCTTTTTCTTAG | 47.6* |  |
| CasRx_F | GGATTCAGCTCCGTCACCAA |  | N/A |
| CaMV-35S_F2 | ACGTAAGGGATGACGCACAA | 56.0 |  |
| TC430 | GTTGGATCTCTTCTGCAGCA | 55.0 | 820 |
| M13F | GTAAAACGACGGCCAGT | 51.7 |  |
| TRV2-confirm_F | CATAATTATACTGATTTGTCTCTCG | 49.4 | N/A |
| RTbent_actin_F | GTCCCTATACGCCAGT | 50.3 | 216**  488*** |
| RTbent_actin_R | ACATCGCGGACAATTT | 48.9 |  |
| *binding Tm  ** cDNA  *** DNA  lower case nts: primer extension sequence | | |  |

**Supplementary Table 3.** RT-qPCR primers used for gene expression analysis.

| **Primer name** | **Sequence 5’-3’** | **Tm (°C)** | **Amplicon (bp)** |
| --- | --- | --- | --- |
| APR_F | CATCAGTGTCGTTGCAGGTATT | 55.3 | 107 |
| APR_R | GCAACTTCTTGGGTTTCCTCAT | 55.6 |  |
| CasRx_gRNA-scaf_F | AACCCCTACCAACTGGTCGGG | 59.9 | ~70 |
| CP_T1_qPCR_R | TAGACCCGCGCGGAAAGTTG | 59.6 |  |
| CP_T2_qPCR_R | CTGAGCTGGTGGGAAGGATGAGA | 59.5 |  |
| CP_T3_qPCR_R | TAGCGAGGGACCCGTGAAAGG | 60.1 |  |
| CasRx_qPCR_2F | TGTTTCCGCCTTCAGCAAAC | 56.1 | 147 |
| CasRx_qPCR_2R | TTAGCGTTGACTCCGATGAGAG | 56.1 |  |
| GVA_qPCR Rep_F | ATCGCGAGCGCAAAAAGTAC | 56.4 | 76 |
| GVA_qPCR Rep_R | TGGCAACGATTTCAGAACGC | 56.2 |  |
| GVA_qPCR CP_F1 | TTGTATCTGAGCTGGTGGGAAG | 55.8 | 87 |
| GVA_qPCR CP_R1 | ACATTTGCCTAAGCGTTGCC | 56.2 |  |
| GVA_qPCR CP_F2 | CGGTACTAGCAAAAAGGCGATC | 55.9 | 139 |
| GVA_qPCR CP_R2 | AGCCACACTCAGAGTTCTCATC | 55.9 |  |
